# Airway Epithelial Inflammation *In Vitro* Augments the Rescue of Mutant CFTR by Current CFTR Modulator Therapies

**DOI:** 10.3389/fphar.2021.628722

**Published:** 2021-03-30

**Authors:** Martina Gentzsch, Deborah M. Cholon, Nancy L. Quinney, Mary E. B. Martino, John T. Minges, Susan E. Boyles, Tara N. Guhr Lee, Charles R. Esther, Carla M. P. Ribeiro

**Affiliations:** ^1^ Marsico Lung Institute and Cystic Fibrosis Research Center, School of Medicine, University of North Carolina, Chapel Hill, NC, United States; ^2^ Department of Pediatrics, Division of Pediatric Pulmonology, School of Medicine, University of North Carolina School of Medicine, Chapel Hill, NC, United States; ^3^ Department of Cell Biology and Physiology, School of Medicine, University of North Carolina School of Medicine, Chapel Hill, NC, United States; ^4^ Department of Medicine, Division of Pulmonary Diseases, School of Medicine, University of North Carolina, Chapel Hill, NC, United States

**Keywords:** airway inflammation, CFTR, F508del, CFTR corrector, CFTR potentiator, CFTR rescue

## Abstract

In cystic fibrosis (CF), defective biogenesis and activity of the cystic fibrosis transmembrane conductance regulator (CFTR) leads to airway dehydration and impaired mucociliary clearance, resulting in chronic airway infection and inflammation. The most common CFTR mutation, F508del, results in a processing defect in which the protein is retained in the endoplasmic reticulum and does not reach the apical surface. CFTR corrector compounds address this processing defect to promote mutant CFTR transfer to the apical membrane. When coupled with potentiators to increase CFTR channel activity, these drugs yield significant clinical benefits in CF patients carrying the F508del mutation. However, processing of CFTR and other proteins can be influenced by environmental factors such as inflammation, and the impact of airway inflammation on pharmacological activity of CFTR correctors is not established. The present study evaluated CFTR-rescuing therapies in inflamed CF airway epithelial cultures, utilizing models that mimic the inflammatory environment of CF airways. Primary bronchial epithelial cultures from F508del/F508del CF patients were inflamed by mucosal exposure to one of two inflammatory stimuli: 1) supernatant from mucopurulent material from CF airways with advanced lung disease, or 2) bronchoalveolar lavage fluid from pediatric CF patients. Cultures inflamed with either stimulus exhibited augmented F508del responses following therapy with correctors VX-809 or VX-661, and overcame the detrimental effects of chronic exposure to the CFTR potentiator VX-770. Remarkably, even the improved CFTR rescue responses resulting from a clinically effective triple therapy (VX-659/VX-661/VX-770) were enhanced by epithelial inflammation. Thus, the airway inflammatory milieu from late- and early-stage CF lung disease improves the efficacy of CFTR modulators, regardless of the combination therapy used. Our findings suggest that pre-clinical evaluation of CFTR corrector therapies should be performed under conditions mimicking the native inflammatory status of CF airways, and altering the inflammatory status of CF airways may change the efficacy of CFTR modulator therapies.

## Introduction

The most common mutation in cystic fibrosis (CF) manifests as deletion of phenylalanine at position 508 in the cystic fibrosis transmembrane conductance regulator (F508del CFTR). F508del CFTR is retained in the endoplasmic reticulum (ER) and degraded by the proteasomal pathway, which prevents it from reaching the apical membrane. In CF airways, lack of apical functional CFTR causes airway dehydration, increases the mucus % solids, and impairs mucociliary clearance, resulting in chronic airway infection and inflammation (Ratjen and Doring 2003; Boucher 2007a).

Basic research paved the road for CFTR-targeting drugs known as CFTR modulators, e.g., correctors that enhance F508del CFTR transfer to the apical membrane, and potentiators that increase CFTR channel activity (Gentzsch and Mall 2018). However, earlier studies indicated the need for improving the efficacy of CFTR modulators. For instance, while the corrector lumacaftor (VX-809) restored F508del maturation and function to ∼15% of wild-type CFTR *in vitro* (Van Goor et al., 2011; Dekkers et al., 2013; Farinha et al., 2013; He et al., 2013; Loo, Bartlett, and Clarke 2013; Okiyoneda et al., 2013; Ren et al., 2013), this drug alone did not significantly improve lung function in early phase clinical trials (Clancy et al., 2012). Adding the potentiator ivacaftor (VX-770) to VX-809, which creates the drug Orkambi, resulted in modest lung function improvements in clinical trials, but only in patients homozygous for F508del (Boyle et al., 2014; Wainwright et al., 2015). Similar outcomes were obtained with tezacaftor (VX-661) plus VX-770, which creates Symdeko (Donaldson et al., 2018). Subsequent triple combination studies adding the next generation correctors elexacaftor (VX-445) or bamocaftor (VX-659) resulted in more impressive clinical responses (Davies et al., 2018; Keating et al., 2018). In fact, the drug Trikafta (VX-445,VX-661, and VX-770) showed substantial efficacy in phase 3 clinical trials (Heijerman et al., 2019; Middleton et al., 2019) and was recently approved by the Food and Drug Administration for the treatment of CF patients aged 12 years and older who have at least one copy of the F508del mutation. While the new triple combination CFTR modulator therapy appears promising, there is still an unmet need to improve the efficacy of CFTR-targeting treatments for patients that carry one or two F508del mutations, who represent ∼ 90% of the CF population. Little is known regarding the mechanisms by which CFTR modulators facilitate the rescue of F508del CFTR and whether the CF airway environment can have an impact on this process.

Because CF patients initiating CFTR modulator therapies suffer from airway inflammation, we recently evaluated the action of CFTR modulators under conditions that recapitulate the inflammatory status of human CF airways. We utilized a pre-clinical model consisting of well-differentiated primary cultures of F508del/F508del human bronchial epithelia (HBE) grown at air-liquid interface (Ribeiro et al., 2005a; Ribeiro et al., 2005b) and exposed to supernatant from mucopurulent material (SMM) harvested from the airways of excised human CF lungs (Ribeiro et al., 2005a; Ribeiro et al., 2005b; Martino et al., 2009; Ribeiro et al., 2009; Abdullah et al., 2018). SMM contains bacterial products, neutrophil factors (e.g., neutrophil elastase, MMP9, cathepsin G, BPI, lysozyme, macrophage and airway epithelial cytokines) (Abdullah et al., 2018), mucins and hundreds of peptides, and purines (Esther et al., 2008); its cytokine composition is reproducible from patient to patient (Abdullah et al., 2018). Because the airway epithelia of CF patients are exposed to multiple inflammatory mediators *in vivo*, the use of SMM is a superior approach to single inflammatory mediators for testing the impact of the CF airway inflammatory milieu on the efficacy of CFTR modulators. We found that SMM enhanced VX-809-increased F508del CFTR-mediated responses to forskolin and VX-770 (Gentzsch et al., 2018). Notably, while the immature ER-residing form of CFTR (band B) was not significantly increased in CF cultures treated with SMM or VX-809, it was significantly increased in response to the simultaneous treatment with SMM and VX-809 (Gentzsch et al., 2018). On the other hand, while the mature band C was formed in F508del cultures treated only with VX-809, SMM drastically increased VX-809-rescued band C (Gentzsch et al., 2018). SMM did not significantly affect the CFTR mRNA levels in presence or absence of VX-809 (Gentzsch et al., 2018), suggesting that its effect is not via up-regulation of F508del transcription or translation.

While these previous studies demonstrated that inflammation increases the efficacy of older-generation CFTR correctors, its impact on next-generation correctors that are components of recently approved triple combination therapy is not known. Furthermore, it is not clear how (and if) the severity of inflammation affects responses to CFTR modulator therapy. To address these key issues, we conducted studies with HBE cells from F508del homozygous CF patients exposed to SMM or bronchoalveolar lavage fluid (BALF) from pediatric CF patients. These pre-clinical models of airway epithelial inflammation were used to evaluate the efficacy of current CFTR modulator double and triple therapies on F508del rescue.

## Results

### Airway Epithelial Inflammation Triggered by SMM or BALF Augments CFTR Correction by VX-661

We have utilized SMM as representative of the CF inflammatory airway milieu; however, since this material is isolated from patients with end-stage lung disease, it represents the airway environment from severe CF phenotypes. To explore whether functional responses of CFTR are influenced by airway inflammation from patients with less severe disease, we utilized BALF from pediatric patients with CF. Both SMM and BALF from CF patients represent the infectious/inflammatory milieu to which the airway epithelia from these patients are exposed. Because the airway epithelia of CF patients are exposed to the combination of all the factors present in SMM or BALF, as opposed to a defined inflammatory factor, the use of SMM and BALF represents a highly translational model to test the impact of the CF airway inflammatory milieu on the efficacy of CFTR modulators.

**FIGURE 1:**
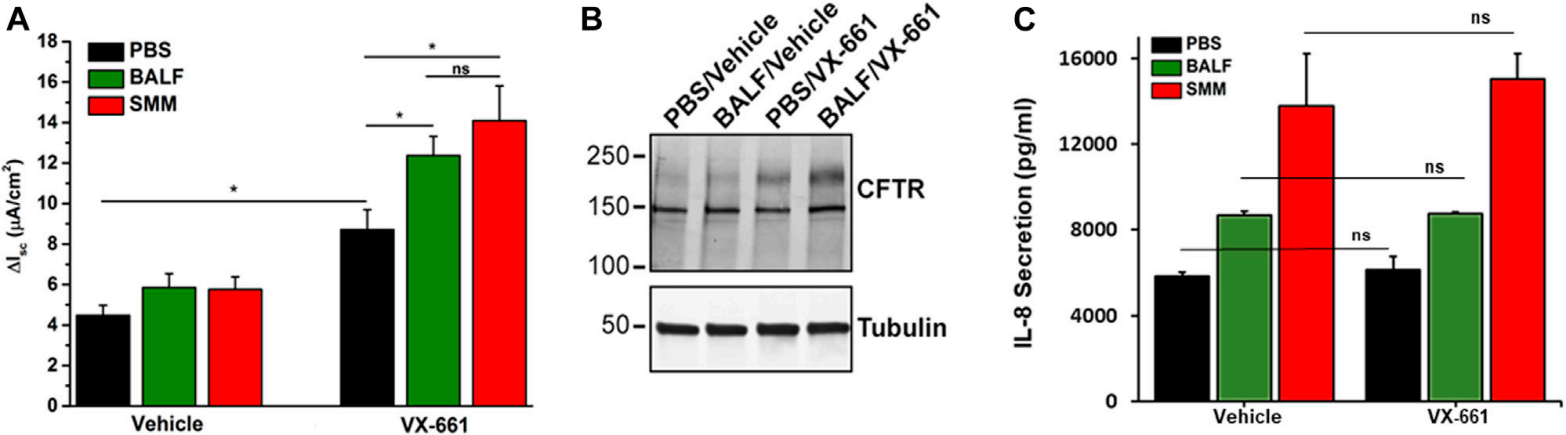
SMM and BALF enhance F508del rescue by VX-661. **(A)** CFTR responses from F508del/F508del HBE evaluated in Ussing chambers. Cultures were exposed apically to 30 µM SMM or BALF and treated on the basolateral side with vehicle or 5 µM VX-661, as described in Methods. Data are expressed as mean ± SEM. *p < 0.05, (t tests), ns = not significant; n = 2 donors, five to six cultures/experimental group. **(B)** Representative CFTR Western blot to analyze F508del maturation (upper band); n = 2 cultures due to limited amounts of BALF. **(C)** IL-8 secretion (pg/ml of culture media) from the F508del/F508del HBE evaluated in **(A)**. Data are expressed as mean ± SEM. Note: Because the BALF samples were diluted, they were concentrated 4-fold with Centricon centrifugal filter units.

We evaluated Symdeko, a CF drug consisting of VX-661 combined with VX-770, which has been approved for patients homozygous for F508del as well as for other genotypes, including partial function CFTR mutations (Rowe et al., 2017; Taylor-Cousar et al., 2017; Donaldson et al., 2018). We tested whether exposure of F508del homozygous primary HBE cultures to BALF from pediatric CF patients or to SMM promoted inflammation coupled with augmentation of VX-661-induced CFTR correction. Similar to VX-809 (Gentzsch et al., 2018), VX-661-induced CFTR correction (as indicated by increased forskolin- and VX-770-induced Cl^−^ secretion) was enhanced by SMM (Figure 1A). Notably, these changes were reproduced in VX-661-rescued cultures exposed to pediatric BALF (Figure 1A). The augmentation of VX-661-promoted functional F508del rescue in BALF-exposed cultures correlated with an enhancement of VX-661-rescued band C levels (Figure 1B; upper-most band), as we have previously shown for SMM in VX-809-treated cultures (Gentzsch et al., 2018). The increased VX-661-mediated CFTR correction in cultures exposed to SMM or BALF was associated with higher levels of epithelial inflammation, based on IL-8 secretory responses (Figure 1C). Importantly, VX-661 did not exhibit an anti-inflammatory action, since it did not blunt the IL-8 secretory response in PBS-, BALF- or SMM-exposed cultures (Figure 1C).

### Airway Epithelial Inflammation Overcomes Chronic VX-770 Treatment-Promoted Inhibition of F508del Rescue

**FIGURE 2:**
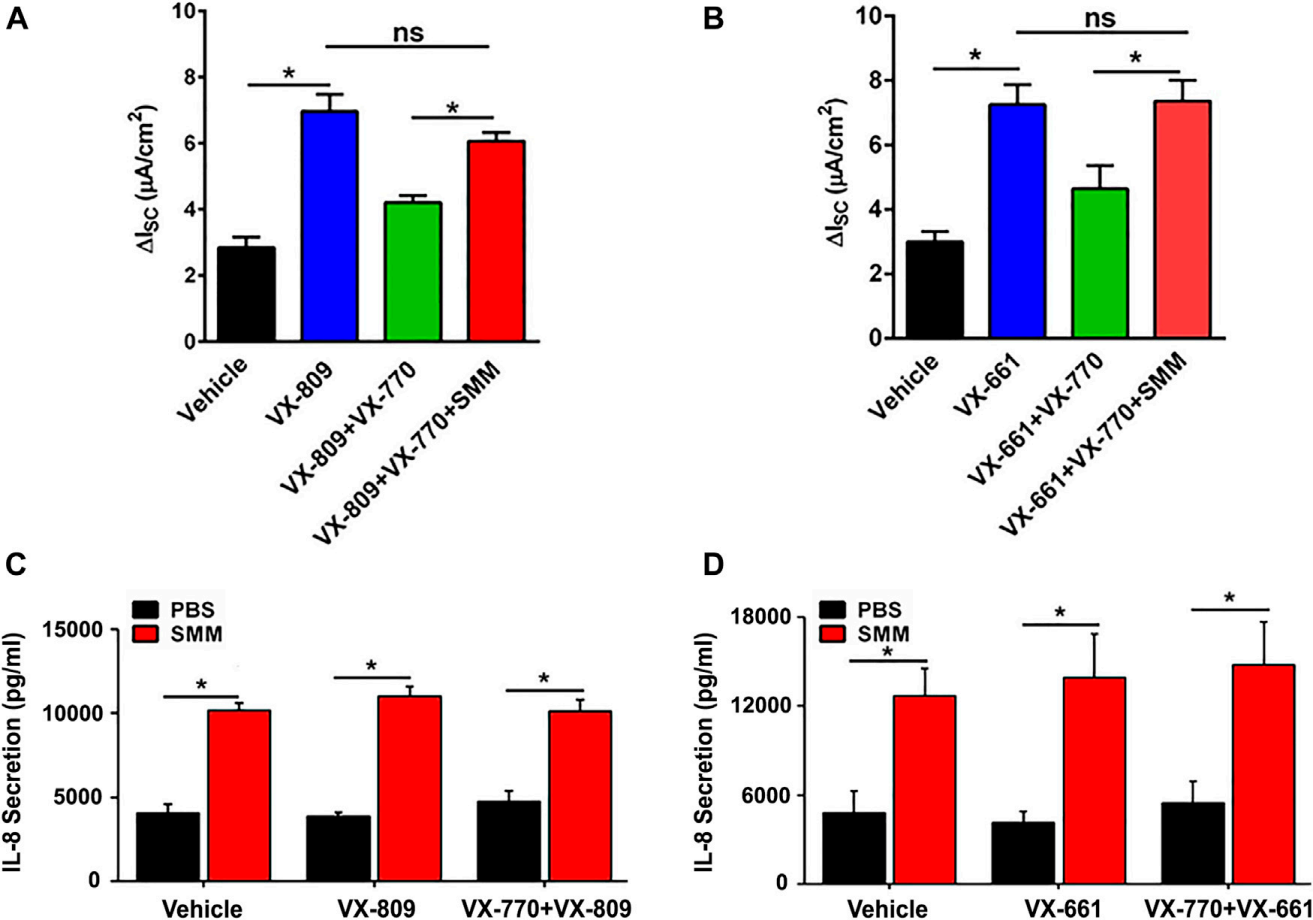
SMM overcomes chronic VX-770 treatment-promoted inhibition of F508del rescue. F508del CFTR responses were measured in Ussing chambers. **(A)** SMM overcomes chronic VX-770 (5 μM, 48 h)-mediated abrogation of VX-809-promoted rescue. **(B)** SMM overcomes chronic VX-770 (5 μM, 48 h)-mediated abrogation of VX-661-dependent rescue. **(C)**,**(D)** IL-8 secretion from F508del/F508del HBE cultures exposed to PBS or SMM and treated for 48 h with VX-809 **(C)** or VX-661 **(D)** ± VX-770. Data are expressed as mean ± SEM. *p < 0.05, ns = not significant; n = 3 CF HBE donors, three cultures/donor.

We have reported that CFTR responses to chronic treatment with VX-809 or VX-661 and VX-770 are lower than when the corrector compound (VX-809 or VX-661) is applied chronically and the potentiator VX-770 is applied acutely (Cholon et al., 2014). In addition, chronic treatment (48 h) of F508de/F508del HBE with VX-770 decreased VX-809-promoted CFTR rescue in a dose-dependent manner (Cholon et al., 2014). We next tested the impact of SMM-induced inflammation regarding the inhibitory effect of chronic VX-770 treatment on VX-809- or VX-661-dependent CFTR rescue. Surprisingly, SMM exposure counteracted chronic VX-770 treatment-induced abrogation of CFTR rescue with either CFTR corrector (Figures 2A,B). We then evaluated the inflammatory responses of the cultures subjected to the various treatments. The baseline and SMM-increased levels of IL-8 secretion were not affected by either corrector, in the absence or presence of VX-770 (Figures 2C,D). Hence, these findings suggest that the increased inflammatory status of F508del HBE cultures resulting from SMM exposure is a common factor for overcoming the detrimental effects of chronic VX-770 treatment on VX-809- or VX-661-mediated CFTR rescue.

### Airway Epithelial Inflammation Overcomes Chronic VX-770 Treatment-Promoted Inhibition of UTP Responses

**FIGURE 3:**
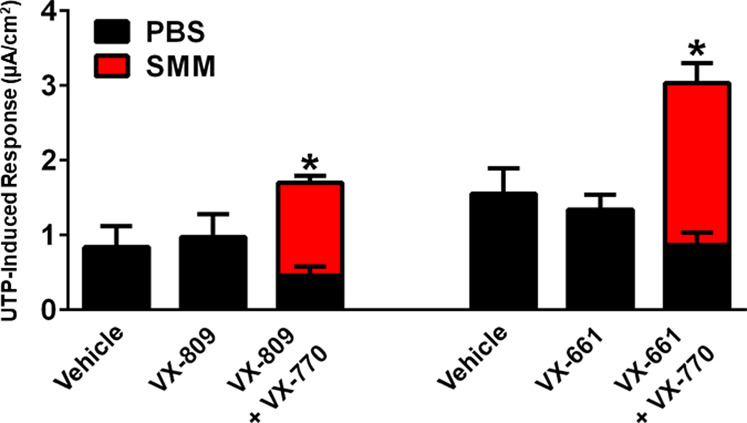
SMM overcomes chronic VX-770 treatment-promoted inhibition of UTP-induced responses. F508del/F508del HBE cultures were exposed for 48 h to vehicle or 5 µM VX-809 or VX-661 ± 5 µM VX-770 in combination with PBS or SMM. Data are expressed as mean ± SEM. *p < 0.05; n = 3 F508del/F508del HBE culture donors, experiments were performed in three replicates/donor.

We have also previously shown that chronic treatment with VX-809 and VX-770 inhibits UTP-induced activation of the calcium-activated chloride channel (CaCC) (Cholon et al., 2014). Therefore, we evaluated whether, similar to its protective action against the detrimental effect of chronic treatment with VX-770 on F508del CFTR, SMM would partially overcome the inhibitory effect of chronic VX-770 treatment on CaCC responses. While the UTP-induced CaCC responses were decreased by chronic treatment of F508del HBE with VX-809 or VX-661 plus VX-770, the inhibitory effect of VX-770 was reversed by exposing the cultures to SMM (Figure 3). Thus, chronic VX-770 treatment targets F508del CFTR and CaCC activities, and our results demonstrate that this inhibition is overcome by inflammation induced by SMM.

### Airway Epithelial Inflammation Enhances Triple Combination-Promoted F508del Rescue

**FIGURE 4:**
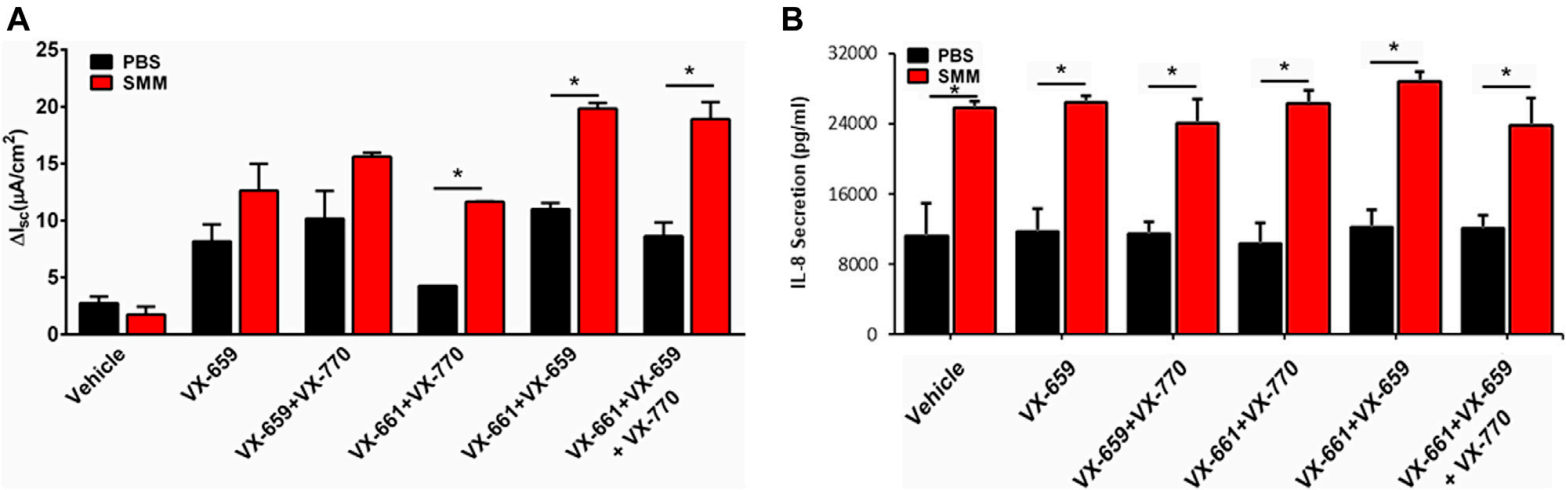
SMM enhances triple combination-promoted F508del rescue. **(A)** CFTR responses to acute forskolin + VX-770 from F508del/F508del HBE in Ussing chambers. HBE were treated with VX-659 (1 µM), VX-661 (5 µM) and VX-770 (5 µM). **(B)** IL-8 secretion from the CF HBE cultures evaluated in **A**. Data are expressed as mean ± SEM. *p < 0.05; n = 2 donors, five to six cultures/experimental group.

Because a triple combination of CFTR modulators (Trikafta consisting of VX-445, VX-661, VX-770) is currently being used in the clinic, we tested the impact of inflammation on the ability of a triple combination therapy consisting of VX-659, VX-661 and VX-770 to increase F508del CFTR rescue. VX-659 is a corrector compound with similar activity as compared to VX-445. In agreement with previous studies (Davies et al., 2018), VX-659, VX-661 and VX-770 promoted 2-fold higher F508del CFTR responses, which approached normal CFTR function, as compared with the responses resulting from the double therapy with VX-661 and VX-770 (Figure 4A). Remarkably, the F508del CFTR responses resulting from the triple combination were further enhanced by SMM by ∼2-fold (Figure 4A). Similar to the findings with other combinations of CFTR modulators (Figures 1C, 2C,D), the triple therapy with VX-659, VX-661, and VX-770 was devoid of an effect on the inflammatory response of the F508del CFTR cultures in the absence or presence of SMM (Figure 4B).

## Discussion

The absence of functional CFTR in CF airway epithelia leads to airway surface liquid dehydration, cilia collapse, and accumulation of thickened mucus to airway surfaces. These alterations result in persistent airway infection and robust chronic inflammation (Doring and Worlitzsch 2000; Saiman and Siegel 2004; Boucher 2007b; Cohen-Cymberknoh et al., 2013). The chronic inflammatory status of CF airways can ultimately damage the airway walls.

Despite decades of research on the cell biology of CFTR, the mechanisms by which CFTR modulators impact rescue of F508del CFTR and the impact of the CF airway environment on this process remain unclear. Utilizing models that more accurately represent the diseased status of CF airway epithelia, our findings demonstrated that the CF airway inflammatory milieu has a major impact on the effect of newer-generation CFTR modulators concerning ion transport processes. Notably, our study has shown that the CF airway inflammatory milieu from advanced CF lung disease (SMM) improves the efficacy of various therapeutic combinations of CFTR modulators regarding their ability to increase F508del CFTR responses (Figures 1, 2, 4). Moreover, similar results were obtained in studies using BALF from pediatric CF patients with less severe disease (Figure 1). Because increased inflammation is already present early in life in CF (Esther et al., 2019), these findings suggest that inflammation-enhanced CFTR rescue is relevant to early CF lung disease. Importantly, inflammation augmented the already impressive CFTR rescue responses from the newly improved triple combination therapy (Figure 4). The integrity of HBE cultures and transepithelial resistance was not affected after SMM or BALF treatment under the experimental conditions utilized (data not shown).

The beneficial effects of inflammation on CFTR rescue by modulator therapy likely reflect inflammation-mediated increases of the ER protein-folding capacity, which is expected to facilitate CFTR folding and trafficking. Short-term F508del CF HBE cultures exhibit an increased expression of ER chaperones and expansion of the ER compartment as a result of their *in vivo* exposure to the CF airway inflammatory milieu; these alterations revert to normal over time in long-term cultures (Ribeiro et al., 2005a). Exposure of long-term cultures of F508del HBE to SMM reproduces the expansion of the ER compartment and its protein folding capacity observed in short-term cultures (Ribeiro et al., 2005a; Ribeiro et al., 2005b; Martino et al., 2009; Ribeiro and O’Neal 2012).

Inflammation also appears to inhibit the destabilization effect of chronic VX-770 exposure on CFTR. We and others have previously demonstrated that chronic treatment with VX-770 abrogates corrector-mediated rescue of F508del by enhancing internalization and turnover of mature CFTR proteins (Cholon et al., 2014; Veit et al., 2014). Exposure to SMM enhanced CFTR responses to chronic treatment with VX-809 or VX-661 plus VX-770 by overcoming chronic VX-770-promoted inhibition of F508del rescue (Figure 2). In agreement with our findings, recent studies have shown that VX-770 can also exert a similar negative effect on the stability of membrane-localized solute carriers (SLC26A3, SLC26A9, and SLC6A14), and it has been suggested that the destabilizing effect of VX-770 may be related to its lipophilicity that may impact the properties of the lipid bilayer (Chin et al., 2018). Future studies are necessary to evaluate whether inflammation of airway epithelia has a similar protective effect against VX-770-induced destabilization of solute carriers and, if so, whether such an effect is due to inflammation-promoted alterations of lipid bilayer properties.

The destabilization effect of VX-770 and the “protective” effect of airway epithelial inflammation were also observed with calcium-activated chloride secretion. UTP-induced CaCC-mediated responses were decreased by chronic treatment with VX-809 plus VX-770 (Figure 3), while exposure to SMM increased UTP-dependent calcium-activated secretion (Figure 3). These effects may further contribute to increased hydration of inflamed CF airways in patients receiving CFTR modulatr therapy. Our findings could potentially be relevant to other chronic lung diseases such as COPD in which VX-770 has been considered as a therapeutic for dysfunctional CFTR (Solomon et al., 2016), and may open new research directions toward understanding the impact of inflammation on biogenesis of other ABC proteins, which are involved in multiple diseases.

Although a recent study suggested that CFTR modulator therapy has anti-inflammatory properties (Jarosz-Griffiths et al., 2020), the experiments performed in our study did not demonstrate anti-inflammatory effects. None of the modulator treatments, including the triple combination, lowered SMM-induced inflammation (indicated by up-regulation of IL-8 secretion), nor lowered the baseline levels of secreted IL-8 (Figures 1, 2, 4). These findings suggest that defective CFTR function (i.e., the “intrinsic” CF defect) is not, per se, a pro-inflammatory condition in airway epithelia, in agreement with our previous studies (Ribeiro et al., 2005b). Airway inflammation in CF likely arises indirectly as a consequence of decreased mucociliary clearance and accumulation of mucus and bacteria. However, we cannot rule out the possibility that CFTR dysfunction contributes to intrinsic inflammatory responses in other cell types.

Furthermore, structural data have indicated that VX-770 directly binds to CFTR protein within membrane domains (Liu et al., 2019). To minimize interference with CFTR modulator interactions, SMM and BALF were added apically, while CFTR modulators were added basolaterally to the culture medium, which should mimic physiological conditions and limit a possible interaction.

**FIGURE 5:**
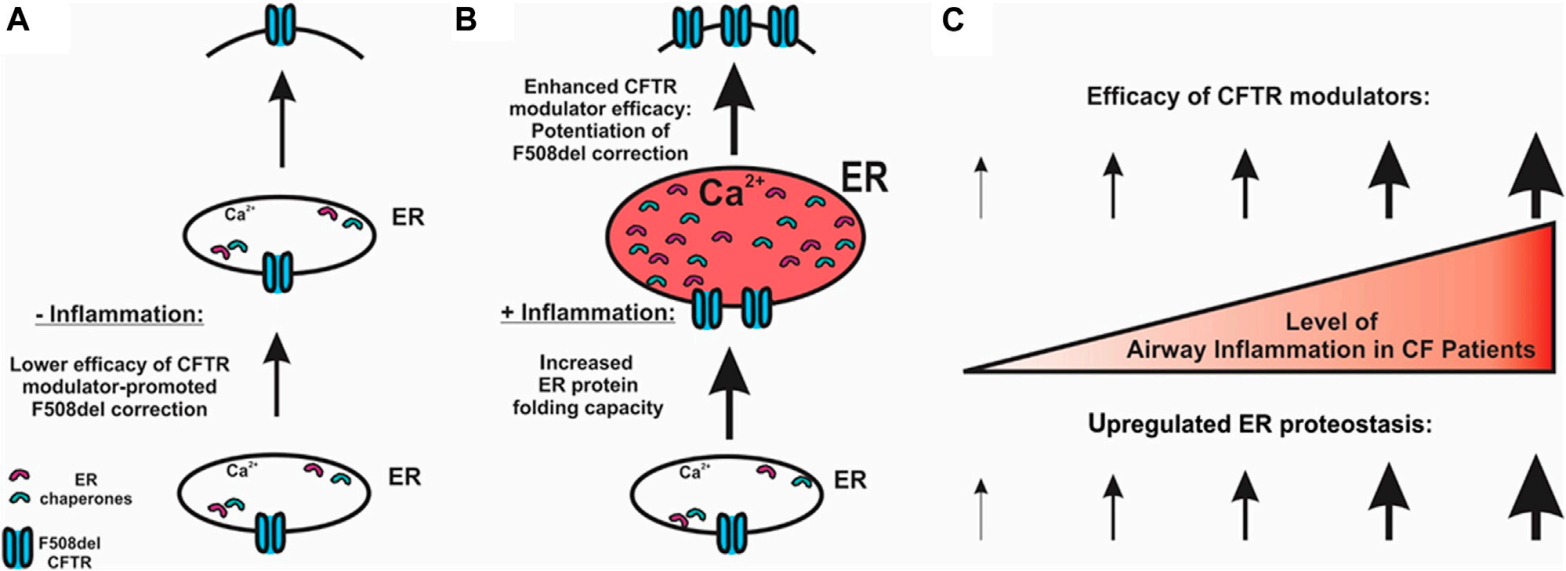
Potential mechanism for airway inflammation-increased efficacy of F508del CFTR modulators. **(A)** In the absence of inflammation, lower expression of chaperones and lower efficacy of CFTR correctors result in a lower level of CFTR folding at the ER. **(B)** CF airway epithelial inflammation increases the ER protein folding capacity, thereby enhancing CFTR modulator efficacy. **(C)** Our model predicts that the higher the levels of airway inflammation, the higher the efficacy of CFTR modulators regarding the rescue of F508del.

The interplay between airway inflammation and responses to CFTR modulators has significant clinical implications. Our findings suggest that the airway inflammation that characterizes CF lung disease plays a major role in the positive clinical responses to CFTR modulator therapy, and we propose a model with a direct relationship between airway inflammation and CFTR rescue (Figure 5). Even the modest inflammation present early in life is likely sufficient to enhance modulator responses. This relationship could limit the clinical efficacy of modulator therapy, since treatment-related improvements in mucociliary clearance could lessen airway inflammation, which would in turn reduce treatment efficacy. Consistent with this hypothesis, two studies demonstrated that highly effective modulator therapy using the CFTR potentiator VX-770 in patients with the G551D mutation did not reduce airway inflammation despite significant improvements in other clinical parameters (Harris et al., 2020; Rowe et al., 2014), although reductions in airway inflammation were observed in a separate study (Hisert et al., 2017). Airway inflammation was not assessed in the clinical trials of the recently approved triple combination modulator therapy, and further studies will be needed to assess the relationships between inflammation and clinical efficacy with this treatment. Although clinicians would not advocate increasing airway inflammation to enhance CFTR modulator efficacy, a better understanding of the underlying mechanisms may allow development of therapies that promote the beneficial effects of inflammation on CFTR rescue without its detrimental consequences.

In summary, the airway inflammatory milieu from late- and early-stage CF lung disease improves the efficacy of CFTR modulators, including triple combination therapy with VX-659, VX-661, and VX-770. Our findings suggest that the balance between suppression of airway inflammation and enhancement of CFTR rescue should be considered in CF patients undergoing anti-inflammatory and CFTR modulator therapies. Furthermore, our findings suggest that pre-clinical evaluation of novel CFTR modulators should be performed under conditions mimicking the native inflammatory milieu found in the airways of CF patients.

## Materials and Methods

### Primary Cell Culture and Drug Treatments

Primary HBE cells from explant lungs from patients homozygous for the F508del, prepared as described previously (Fulcher and Randell 2013), were obtained from Dr. Scott H. Randell (Marsico Lung Institute, The University of North Carolina at Chapel Hill, USA). The cells were obtained under protocols approved by the University of North Carolina at Chapel Hill Biomedical Institutional Review Board. Cells were expanded in BEGM (Lonza) and then cultured at air-liquid interface on 12 mm Millicell inserts (Millipore) in a modified BEBM (Hild and Jaffe 2016) until differentiated. Well-differentiated F508del/F508del HBE cultures were exposed for 24 h to apically added PBS or pooled SMM or BALF, in combination with basolateral exposure to vehicle or CFTR modulators ivacaftor (VX-770, Selleck Chemicals; 5 µM), lumacaftor (VX-809, Selleck Chemicals; 5 µM), tezacaftor (VX-661, Selleck Chemicals; 5 µM) and bamocaftor (VX-659, a gift from Henry Danahay; 1 µM). For chronic exposure, F508del HBE cultures were treated for 48 h.

### CF SMM and BALF

SMM was obtained from the airways of excised human CF lungs as previously described (Ribeiro et al., 2005b; Martino et al., 2009; Ribeiro et al., 2009; Abdullah et al., 2018) and provided by the UNC CF Center Tissue Procurement and Cell Culture Core. BALF from pediatric CF patients <18 years old, which reflect the inflammatory airway milieu of the population ultimately targeted for mutant CFTR rescue, was pooled and concentrated 4-fold.

### Transepithelial Ion Transport

Changes in short-circuit current (ΔI_sc_) were measured from CF-HBE cultures in Ussing chambers as previously described (Cholon et al., 2014; Gentzsch et al., 2016; Gentzsch et al., 2017) in a bilateral Krebs bicarbonate-Ringers solution. Amiloride (100 µM, Sigma-Aldrich) was added to the apical bath to inhibit the epithelial sodium channel, ENaC. Bilateral addition of forskolin (10 µM, Sigma-Aldrich) followed to stimulate CFTR channel activity. CFTR inhibitor-172 (10 μM, Sigma-Aldrich) was then apically introduced to inhibit CFTR. Transepithelial resistance (Ω cm^2^) was measured to assess monolayer integrity and only cultures with values larger than 100 Ω cm^2^ were utilized. UTP (100 μM, GE Healthcare) response (an index of calcium-activated chloride channel activity) was assessed as an internal control.

### CFTR Western Blotting

Western blot analysis of endogenous CFTR protein was performed as described previously (Cholon et al., 2014; Gentzsch et al., 2016). Briefly, whole-cell lysates of fully differentiated CF HBE cultures were prepared and then CFTR was immunoprecipitated. Samples were separated on 4–20% gradient SDS–polyacrylamide gel electrophoresis gels (Bio-Rad) and then transferred to nitrocellulose. Blots were probed with mouse monoclonal anti-CFTR antibodies and then with IRDye–goat anti-mouse immunoglobulin G (Molecular Probes). Anti-tubulin (LI-COR) was used as a loading control. Protein bands were visualized using an Odyssey Infrared Fluorescence Imaging System (LI-COR).

### SMM (or BALF)-Induced Inflammation

F508del/F508del HBE were exposed to 30 µl mucosal SMM or BALF; serosal media were collected at the end of the experimental protocol to assess IL-8 secretion (an indicator of inflammation) by ELISA, as we have reported (Ribeiro et al., 2005b; Martino et al., 2009).

### Statistical Analysis

Data analysis was performed using GraphPad PRISM software. Data were subjected to ANOVA analysis. Following significant ANOVA results, statistical significance between groups was calculated using Student’s t-test. *p* ≤ 0.05 was considered statistically significant.

## Data Availability

Further inquiries related to the original contributions presented in the study can be directed to the corresponding author.
